# Peptide-MHC-I from Endogenous Antigen Outnumber Those from Exogenous Antigen, Irrespective of APC Phenotype or Activation

**DOI:** 10.1371/journal.ppat.1004941

**Published:** 2015-06-24

**Authors:** Janet J. Sei, Scott Haskett, Lauren W. Kaminsky, Eugene Lin, Mary E. Truckenmiller, Clifford J. Bellone, R. Mark Buller, Christopher C. Norbury

**Affiliations:** 1 Department of Microbiology and Immunology, College of Medicine, Pennsylvania State University, Hershey, Pennsylvania, United States of America; 2 Department of Molecular Microbiology and Immunology, Saint Louis University Health Sciences Center, St. Louis, Missouri, United States of America; La Jolla Institute for Allergy and Immunology, UNITED STATES

## Abstract

Naïve anti-viral CD8^+^ T cells (T_CD8+_) are activated by the presence of peptide-MHC Class I complexes (pMHC-I) on the surface of professional antigen presenting cells (pAPC). Increasing the number of pMHC-I *in vivo* can increase the number of responding T_CD8+_. Antigen can be presented directly or indirectly (cross presentation) from virus-infected and uninfected cells, respectively. Here we determined the relative importance of these two antigen presenting pathways in mousepox, a natural disease of the mouse caused by the poxvirus, ectromelia (ECTV). We demonstrated that ECTV infected several pAPC types (macrophages, B cells, and dendritic cells (DC), including DC subsets), which directly presented pMHC-I to naïve T_CD8+_ with similar efficiencies *in vitro*. We also provided evidence that these same cell-types presented antigen *in vivo*, as they form contacts with antigen-specific T_CD8+_. Importantly, the number of pMHC-I on infected pAPC (direct presentation) vastly outnumbered those on uninfected cells (cross presentation), where presentation only occurred in a specialized subset of DC. In addition, prior maturation of DC failed to enhance antigen presentation, but markedly inhibited ECTV infection of DC. These results suggest that direct antigen presentation is the dominant pathway in mice during mousepox. In a broader context, these findings indicate that if a virus infects a pAPC then the presentation by that cell is likely to dominate over cross presentation as the most effective mode of generating large quantities of pMHC-I is on the surface of pAPC that endogenously express antigens. Recent trends in vaccine design have focused upon the introduction of exogenous antigens into the MHC Class I processing pathway (cross presentation) in specific pAPC populations. However, use of a pantropic viral vector that targets pAPC to express antigen endogenously likely represents a more effective vaccine strategy than the targeting of exogenous antigen to a limiting pAPC subpopulation.

## Author Summary

To induce a protective cell type (CD8+ T cells) following virus infection, it is necessary to present degraded fragments of viral protein in complex with self molecules on the surface of so-called antigen presenting cells (APC). This process can occur in infected or uninfected APC and has been studied and quantified extensively in experimental setups in the lab. However, the extent to which presentation by infected or uninfected cells contribute to the induction of a protective CD8+ T cell response has not been studied extensively during a natural infection in a mouse model. Here we use a natural mouse virus to examine importantly, quantify, the contribution of presentation of the fragments of viral protein by infected or uninfected cells. We find that the presentation by infected cells dwarfs that seen by uninfected cells. The importance of this work lies in the fact that, if infected cells present way more antigen than uninfected cells, successful vaccine design should utilize this observation to make a vaccine where infected cells expressing virus proteins are the prevalent mode of induction of CD8+ T cells.

## Introduction

In the fight against virus invasion, T_CD8+_ play an essential role by killing virus-infected cells. Activation of these cells by professional antigen presenting cells (pAPC) is a vital step in generation of an effective adaptive immune response. pAPC are a heterogeneous population comprised of B cells, macrophages and dendritic cells (DC), and specialized subsets of each of those populations. Numerous studies have examined the abilities of these populations and subpopulations to present pMHC-I following virus infection or immunization [1–6]. These studies have concluded that certain pAPC populations are specialized for particular functions, leading to multiple strategies targeting particular pAPC populations in vaccine design [7]. However, the extent to which pAPC populations provide sufficient pMHC-I for maximal generation of T_CD8+_ depends on factors such as viral tropism for pAPC populations [8], interference with pMHC-I processing pathways [9], or lysis of infected pAPC populations [10].

To date, previous studies have relied upon the semi-quantitative activation of T cells, measured either by initiation of proliferation or acquisition of effector functions such as cytokine production or lytic activity. Each measure of T cell activity is quantitative only in the sense that each T cell has undergone proliferation or displayed effector activity, but these activities are affected by many other factors, including the expression of costimulatory and adhesion molecules by T_CD8+_ or pAPC, the cytokine milieu and/or modulation of each of these factors by virus infection or pre-activation of the pAPC by other inflammatory stimuli [11]. Here we have quantitatively examined antigen presentation following infection with a poxvirus, the natural mouse pathogen ectromelia virus (ECTV), which is pantropic for all pAPC populations examined. Our system allowed us to differentiate between presentation of endogenously synthesized antigen by multiple populations of infected pAPC (direct presentation) and presentation of antigen acquired by uninfected pAPC populations (cross presentation). We have demonstrated that presentation of endogenously synthesized antigen results in much higher pMHC-I levels than acquisition of exogenous antigen and that, on a per cell basis, each infected pAPC population produces equivalent pMHC-I levels, irrespective of activation or maturation status. These data have important ramifications for rational vaccine design in that they indicate that a vaccine in which endogenous synthesis of the targeted antigen occurs within multiple pAPC populations is the most effective way to generate the greatest number of effective pMHC-I complexes which, in turn, results in an optimal antigen specific T_CD8+_ response.

## Results

### ECTV infects lymphoid cells *in vivo*, and ECTV-infected cells directly present SIINFEKL on K^b^ in a TAP dependent manner

**Fig 1:**
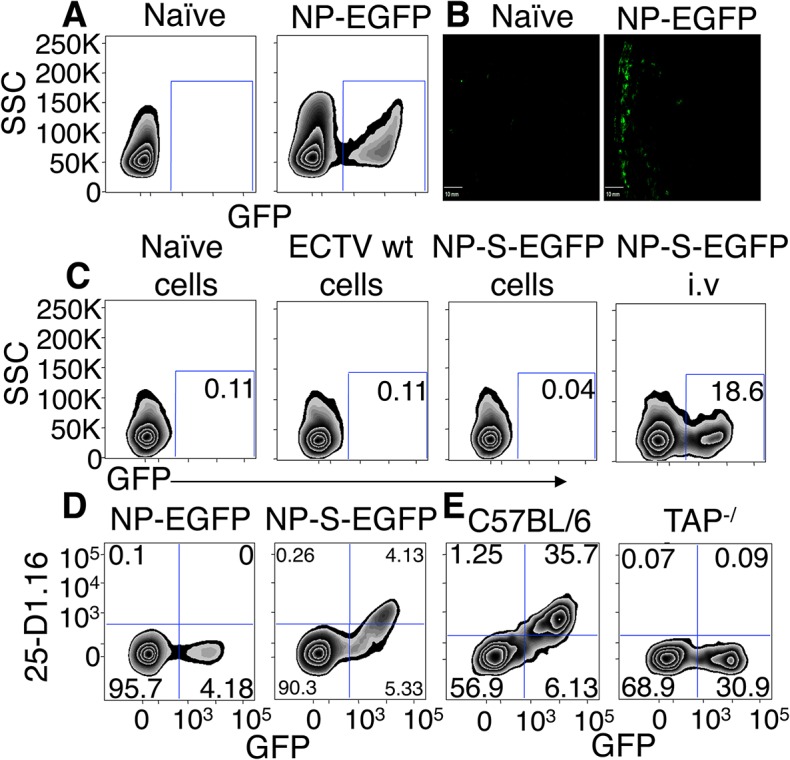
EGFP^+^ cells are infected by ECTV and directly present antigen in a TAP dependent manner. (A and B) Expression of EGFP 12 h.p.i with NP-EGFP i.d. or vehicle. D-LN were analyzed by flow cytometry (12 h.p.i) (A) or by fluorescence microscopy (6 h.p.i) (B) (Representative of 5 experiments). (C) C57BL/6.SJL cells were infected with ECTV wt or NP-S-EGFP *in vitro*, treated with UV-C and psoralen and injected i.v. into C57BL/6 mice. Positive control C57BL/6 mice were injected i.v. with ECTV NP-S-EGFP. Twelve hours later, spleens were harvested and recipient cells were analyzed for EGFP expression by flow cytometry (Representative of 3 experiments. Nos. are % of cells). (D) Expression of K^b^-SIINFEKL complexes by splenocytes 24 hr after immunization with NP-S-EGFP or NP-EGFP i.v. analyzed by flow cytometry (Representative of >10 experiments (n = 3 mice per condition per experiment) and nos. represent % of cells). (E) TAP1^-/-^ or C57BL/6 mice were injected with NP-S-EGFP, as described in (D) (Representative of 3 experiments and nos. represent % of cells).

To quantify ECTV infection and subsequent antigen presentation, we utilized a recombinant ECTV virus that encodes a fusion protein (NP-S-EGFP) consisting of the influenza nucleoprotein (NP), an enhanced green fluorescent protein (EGFP), and ovalbumin (OVA) residues 257–264 (SIINFEKL) [12]. This system allows us to identify ECTV-infected and uninfected cells based on the presence and absence of EGFP expression. Following injection with NP-S-EGFP i.d., draining lymph nodes (D-LN) were harvested at 12 h.p.i. from naïve or ECTV-infected mice. A distinct EGFP^+^ cell population was observed (Fig 1A). ECTV-infected cells were resident in the periphery of the D-LN, just below the sub-capsular sinus by 6 h.p.i (Fig 1B). To assess whether the EGFP^+^ cells were infected by ECTV and were not uninfected cells that had engulfed dead or dying EGFP^+^ cells or EGFP^+^ cellular material, we conducted the following experiment. Splenocytes from C57BL/6.SJL (CD45.1^+^) mice were infected *in vitro* with ECTV NP-S-EGFP or wild type (wt) ECTV to allow expression of viral antigen and then treated with psoralen and UV-C-crosslinking to abolish further virus replication [13] (S1A Fig). The infected and psoralen/UV treated cells were injected i.v into C57BL/6 (CD45.2^+^) mice, and spleens subsequently assessed for the presence of recipient-derived EGFP^+^ cells. As a positive control, mice were directly infected i.v with a dose of NP-S-EGFP that was 30-fold lower than the number of infected splenocytes injected. We found EGFP^+^ cells in mice directly infected with ECTV NP-S-EGFP but not in naïve mice or mice immunized with either WT ECTV or a large excess of NP-S-EGFP-infected cells (Fig 1C). Notably, infection of cells by ECTV *in vivo* was dependent on virus replication (S1A Fig). These results demonstrate that EGFP^+^ cells resulted from ECTV infection, and not from internalization of EGFP^+^ material by uninfected cells.

We isolated cells from the D-LN of mice infected with ECTV NP-S-EGFP or NP-EGFP (which lacks the OVA_257-264_ SIINFEKL determinant) 12 h.p.i. and stained with an antibody specific for K^b^-SIINFEKL complexes [14]. Cells from mice inoculated with ECTV NP-EGFP did not show staining above background. Infected cells from ECTV NP-S-EGFP-infected mice expressed measurable levels of K^b^-SIINFEKL complexes (Fig 1D) but none of the uninfected GFP^-^ cells from mice infected with ECTV NP-S-EGFP displayed antibody staining (Fig 1D). To ensure that antigen presentation in infected cells occurred via the conventional endogenous processing pathway, we measured antigen presentation following infection of mice lacking TAP1, a vital component of this pathway. Mice lacking TAP1 did not display staining for K^b^-SIINFEKL complexes above background levels (Fig 1E). Collectively, these results indicate that this infection allows differentiation between virus-infected and uninfected cells *in vivo* and accurate quantification of specific peptide-MHC complexes on infected cells.

### DC, B cells and macrophages are infected by ECTV and directly present antigen that leads to priming of naïve T_CD8+_

**Fig 2:**
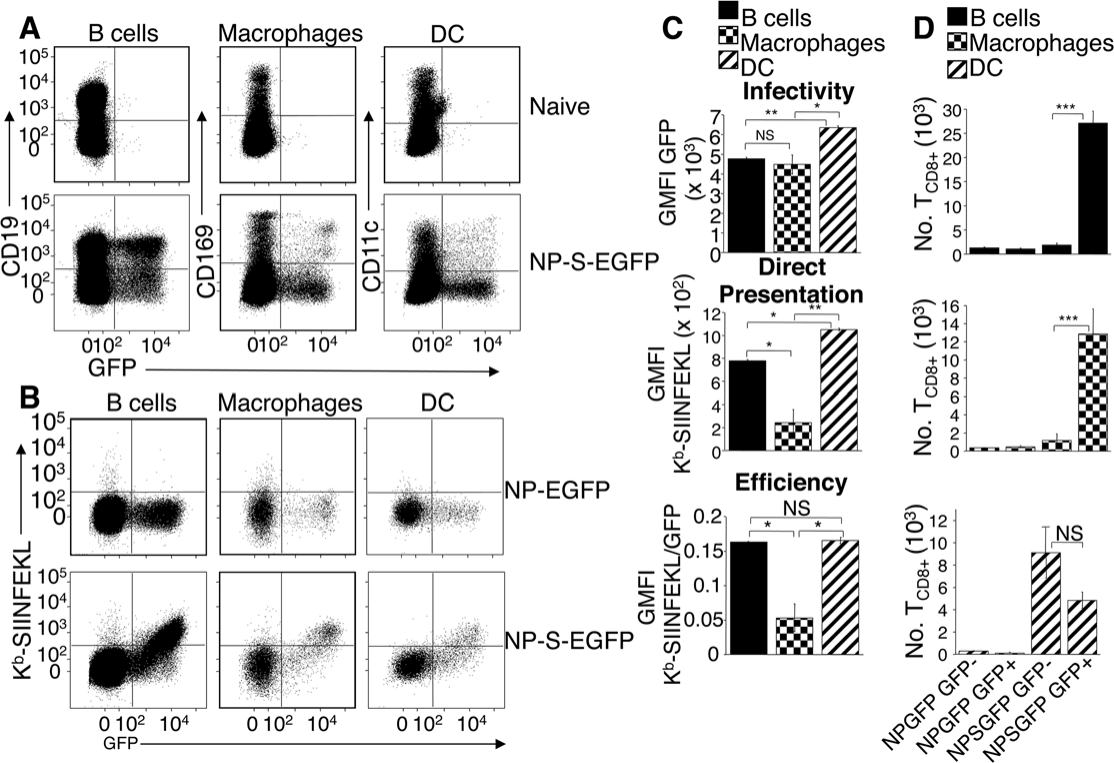
Dendritic cells, B cells, and macrophages are infected by ECTV and stimulate naïve OT-I T_CD8+_. (A) Mice were injected with NP-S-EGFP, as in Fig 1D. pAPC analyzed for EGFP expression were identified as B cells (CD19^+^ CD11c^-^ CD169^-^), macrophages (CD169^+^ CD11c^-^ CD19^-^), and DC (CD11c^+^ CD169^-^ CD19^-^) (Representative of 5 experiments, n = 3 mice per condition per experiment). See also S1B Fig for gating strategy. (B) As in A with addition of anti-K^b^-SIINFEKL antibody. (C) Graphical quantification of data from A & B. Expression of K^b^-SIINFEKL complexes was quantified by subtracting NP-EGFP geometric mean fluorescence intensity (GMFI) of anti-K^b^-SIINFEKL antibody from NP-S-EGFP. Efficiency of direct presentation was calculated as the ratio of GMFI K^b^-SIINFEKL/GMFI EGFP (D) Stimulation of naive OT-I T_CD8+_ by pAPC. Twenty four h.p.i with NP-EGFP or NP-S-EGFP D-LN were sorted for EGFP^+^ or EGFP^-^ B cells, macrophages or DC (see above). Sorted cells were co-cultured separately with naïve T_CD8+_ from OT-I.SJL mice for 60 hr, and proliferation of T_CD8+_ analyzed. Data in (C) and (D) are pooled from 3 independent experiments (mean ± standard error, n = 3 mice per condition per experiment). P values *p<0.05, **p<0.01, ***p<0.001, NS (not significant), student’s unpaired t-test.

To examine the pAPC (DC, B cells and macrophages) infected by ECTV, we injected vehicle, NP-EGFP, or NP-S-EGFP i.d., and harvested D-LN at 24 h.p.i. We stained with a panel of antibodies to identify DC (CD11c^+^ CD169^-^ CD19^-^), B cells (CD19^+^ CD11c^-^ CD169^-^ B220^+^), and macrophages (CD11b^+^ CD11c^-^ CD19^-^ CD169^+^) (S1B Fig). A kinetic analysis indicated that CD169^+^ macrophages were the first pAPC to be infected, while CD19^+^ B cells and CD11c^+^ DC were infected by 12 h.p.i. (S2A Fig). Therefore, at 24 h.p.i all major populations of pAPC were infected (S2A Fig), allowing us to compare the efficiency of antigen presentation by each pAPC population. We compared the fluorescence produced from antigen-conjugated GFP in each pAPC population (Fig 2B). B cells and macrophages expressed equivalent levels of antigen, but DC expressed significantly more ECTV-encoded antigen on a per cell basis (Fig 2C, top panel). As above, we found that only infected pAPC stained for K^b^-SIINFEKL. Staining of uninfected B cells, macrophages and DC was indistinguishable from cells isolated from mice infected with control ECTV-NP-GFP. We found higher levels of K^b^-SIINFEKL complexes on the surface of DC than on the surface of B cells, and each was significantly higher than the levels observed on the surface of macrophages (Fig 2C middle panel). The levels of K^b^-SIINFEKL complexes increased with time after infection with NP-S-EGFP (S2B Fig). Because DC express more ECTV antigen than B cells or macrophages (Fig 2C, top panel) we sought to ascertain the efficiency of antigen presentation in each pAPC population by equalizing for protein expression. Therefore, we calculated the efficiency of direct presentation as a ratio of K^b^-SIINFEKL complexes per EGFP protein, which represents the formation of pMHC-I complexes as a function of the levels of the protein antigen from which the complexes were derived. DC and B cells were equally efficient at producing K^b^-SIINFEKL complexes while macrophages were significantly less efficient (Fig 2C, bottom panel).

Although K^b^-SIINFEKL complexes were only detected on the surface of infected pAPC populations, levels below the threshold of detection with the 25.D1.16 antibody might still be capable of T_CD8+_ stimulation [14]. Therefore, we analyzed the ability of ECTV-infected and uninfected pAPC populations to activate naïve SIINFEKL-specific OT-I T_CD8+_ [15]. Mice were injected in the footpads with either NP-EGFP or NP-S-EGFP, and EGFP^+^ or uninfected EGFP^-^ B cells, DC and macrophages were sorted from D-LN cell suspensions. Each cell population was co-cultured separately with naïve OT-I T_CD8+_ and T_CD8+_ proliferation was determined at 60 h post-culture. None of the pAPC populations purified from mice infected with control NP-EGFP, activated OT-I T_CD8_ above background (Fig 2D). Only NP-S-EGFP-infected B cells and macrophages robustly activated naive OT-I T_CD8+_, whereas uninfected B cells and macrophages did not stimulate naive OT-I T_CD8+_ (Fig 2D). Notably, both ECTV-infected and uninfected DC were capable of activating naïve OT-I T_CD8+_ (Fig 2D bottom panel). Thus, although K^b^-SIINFEKL complexes were undetectable with antibody staining on EGFP^-^ DC (Fig 1D and 2B), these uninfected DC appear specialized (compared to B cells and macrophages) to express sufficient K^b^-SIINFEKL complexes to stimulate the high affinity TCR on OT-I T_CD8+_.

### Antigen specific T_CD8+_ relocate to peripheral regions of D-LN after infection with NP-S-EGFP and interact with infected pAPC expressing cognate antigen

**Fig 3:**
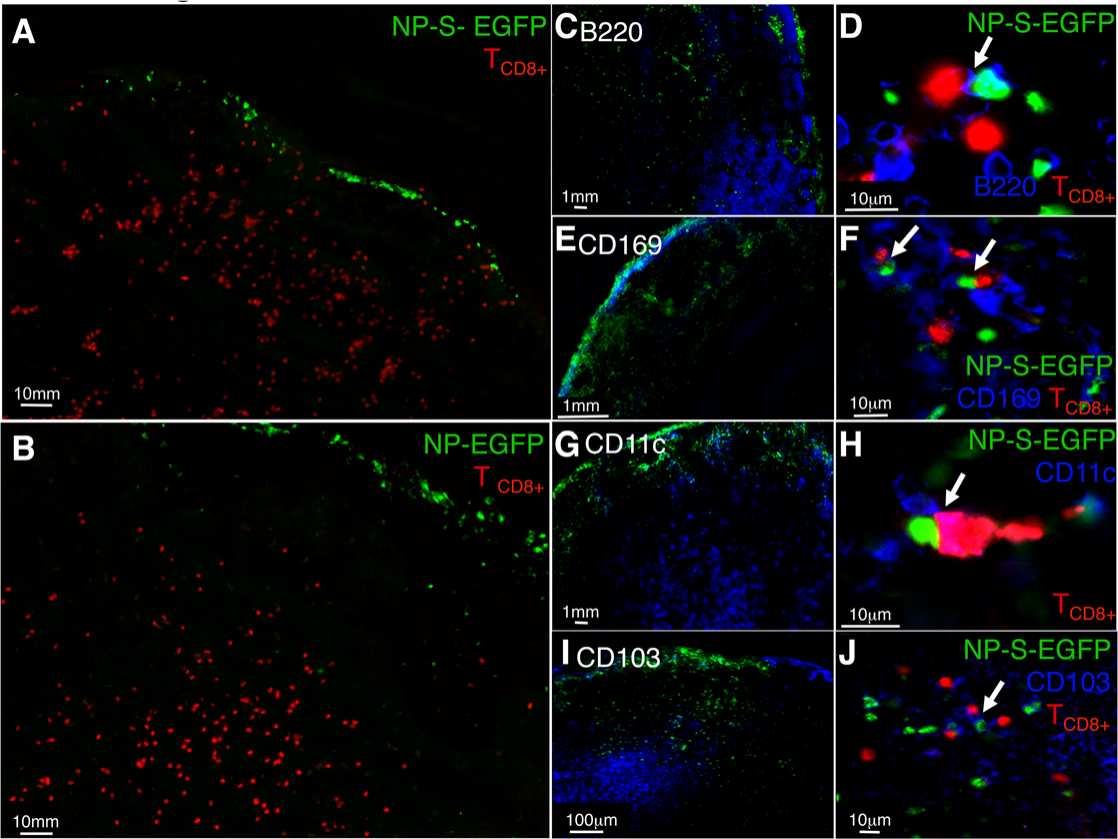
Antigen specific T cells relocate to the LN periphery where they interact with infected pAPC expressing antigen *in vivo*. (A and B) Localization of OT-I T_CD8+_ following ECTV infection. Naïve OT-I T_CD8+_ were labeled with cell tracker CMTMR dye (red) and adoptively transferred. Mice were injected with NP-S-EGFP or NP-EGFP i.d., and 12 h.p.i., D-LN were harvested and analyzed by fluorescence microscopy. (C, E, G, I) Mice were injected with NP-S-EGFP or NP-EGFP i.d., and 24 h.p.i. D-LN were harvested and stained for B220^+^ B cells, CD169^+^ macrophages, CD11c^+^ DC and CD103^+^ DC, and analyzed by fluorescence microscopy. (D, F, H, J) High power view of interaction of naïve OT-I T_CD8+_ and ECTV-infected pAPC. The insets (D, F, H, and J) show 2-dimensional projections of one plane of the 3-dimensional datasets. Each image is representative of 3 experiments, with a minimum of 4 infected nodes per experiment.

Although we demonstrated antigen presentation by all infected pAPC populations, it was not clear whether all infected pAPC populations are located at sites at which naïve T_CD8+_ are activated. Therefore, we visualized the interaction of labeled naïve OT-I T_CD8+_ with virus-infected pAPC expressing cognate antigen. Recipient mice were injected with either NP-EGFP or NP-S-EGFP i.d., and at 12 h.p.i (Figs 3A and 3B) or 24 h.p.i. (Figs 3C–3J), D-LN were harvested for microscopic analysis. ECTV-infected cells were predominantly located at the periphery of the D-LN just below the sub-capsular sinus at early time points, with a few cells observable in the cortical region (Figs 3A and 3B), as we [16] and others [17] have previously described following infection with the related poxvirus vaccinia virus (VACV). However, in contrast to short–lived VACV infection, where the number of GFP+ cells is reduced over time [16], following ECTV infection, EGFP^+^ cells were visualized 300 m from the periphery at 24 h.p.i, and the number of infected cells had increased significantly (Figs 3C, 3E, 3G and 3I), mirroring our flow cytometry analyses (S2A Fig). Notably, in D-LN infected with SIINFEKL-expressing virus (NP-S-EGFP), the OT-I T_CD8+_ relocated into the peripheral regions of the D-LN (Fig 3A), presumably, to interact with virus-infected cells. However, in D-LN infected with NP-EGFP (Fig 3B), OT-I T_CD8+_ were restricted to the T cell zone.

To determine the interaction of individual pAPC populations with naïve T_CD8+_, cryosections were stained with anti-B220 (B cells), anti-CD169 (macrophages), anti-CD11c (DC), or anti-CD103 (migrating DC) antibodies and visualized by fluorescence microscopy. As expected, we primarily observed B220^+^ cells in the B cell follicle region (Fig 3C), CD169^+^ in the sub-capsular region (Fig 3E), and CD11c^+^ or CD103^+^ cells in the cortical region of the D-LN (Figs 3G and 3I). To visualize direct interaction between OT-I T_CD8+_ and ECTV-infected pAPC, we acquired and analyzed 3-dimensional high power images. When analyzing the images produced we considered that there would not be direct co-localization of cell surface stain with GFP, which is localized within the nucleus as it is attached to NP. In D-LN from mice infected with NP-S-EGFP, we visualized OT-I T_CD8+_ interacting with EGFP^+^CD169^+^ macrophages (Fig 3F), EGFP^+^CD11c^+^ DC (Fig 3H), EGFP^+^CD103^+^ DC (Fig 3J) and, surprisingly, EGFP^+^B220^+^ B cells (Fig 3D) within 24 h of infection. Therefore, the antigen presentation that we measured *in vitro* by each pAPC population in Fig 2 has the potential *in vivo* to induce the activation of naïve T_CD8+_.

### CD11b^+^ DC, CD8α^+^ DC, and pDC subsets are infected by ECTV and directly present antigen on MHC class I with equivalent efficiency

**Fig 4:**
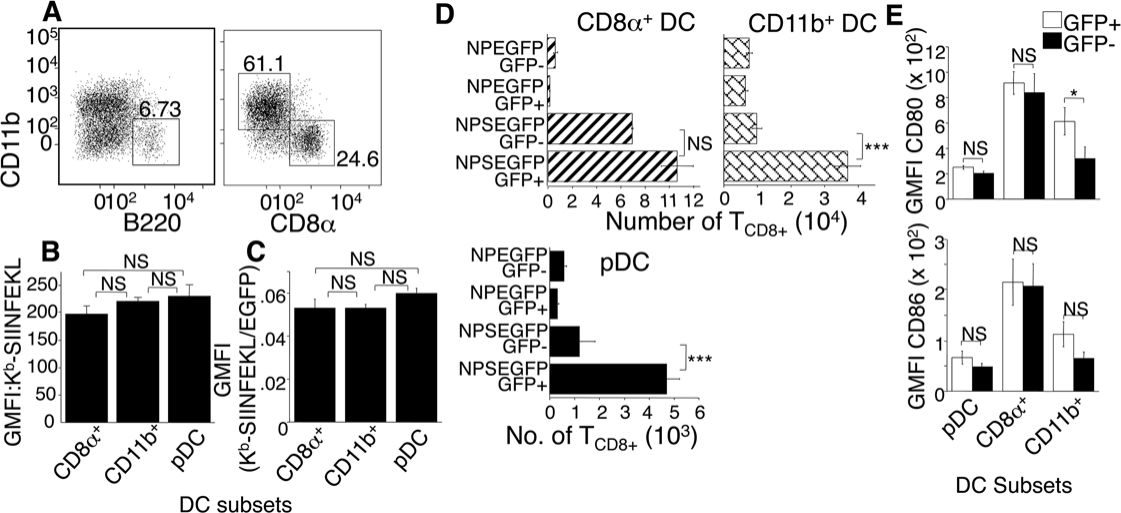
DC subsets are equally efficient in direct antigen presentation. (A) Mice were injected with ECTV, D-LN harvested and cells stained with antibodies to identify non-NK, non-B, non T cell, GFP^+^ CD11c^+^ DC subsets as: pDC (B220^+^CD11b^-^), CD8α (B220^-^CD8α ^+^CD11b^-^), CD11b^+^ (B220^-^CD11b^+^CD8α ^-^). Nos. represent % of cells in 3 representative experiments using 3 mice per condition. (B and C) Mice were injected with NP-S-EGFP or NP-EGFP, D-LN harvested and stained as described in (A), with the addition anti- K^b^-SIINFEKL. Quantification of K^b^-SIINFEKL expression and efficiency was determined as described in Fig 2C. Data are pooled from 3 experiments using 3 mice per condition. (D) Mice were injected with NP-EGFP or NP-S-EGFP, then D-LN cells were FACS sorted for EGFP^+^ or EGFP^-^ pDC, CD8α ^+^, or CD11b^+^ DC, as above. Each population was co-cultured with OT-I T_CD8+_ that were then analyzed for proliferation as above. Data are pooled from 3 experiments, using 15 mice per condition to obtain sufficient cells. (E) As in (A), except for addition of anti-CD80 and anti-CD86 antibodies. Data are pooled from 3 experiments. All graphs show (mean ± standard error), P values *p<0.05, **p<0.01, ***p<0.001, NS (not significant) using Student’s unpaired t-test.

DC are composed of different subpopulations that are proposed to be specialized to perform differing tasks during antigen presentation [5]. Several studies have reported a role for individual DC subsets in MHC class I mediated T_CD8+_ activation [1–3, 5, 6]. However, during a virus infection it is vital to account for viral tropism for individual DC subsets versus functional specialization of DC presenting viral antigen. We focused on the three major DC subsets in lymph node and spleen characterized as: CD8α^+^ CD11b^-^ B220^-^ (hereafter CD8α^+^ DC), CD11b^+^ CD8α ^-^ B220^-^ (hereafter CD11b^+^ DC), and plasmacytoid B220^+^ CD11b^-^ (hereafter pDC). To determine whether there is specialization in MHC class I presentation by infected DC subsets, mice were injected with NP-EGFP or NP-S-EGFP i.d., and D-LN were harvested at 24 h.p.i. Cells were stained to identify DC subsets and analyzed by flow cytometry. As NK cells, T cells and B cells share some DC markers and may alter antigen presentation [6] we stained with antibodies to identify NK cells, T cells and B cells, to exclude these lymphoid populations from our analysis. We found that GFP+ cells contained all DC subsets (Fig 4A). We did not observe staining for K^b^-SIINFEKL complexes on any uninfected cell population. The number of K^b^-SIINFEKL complexes on the surface (Fig 4B) and efficiency with which these K^b^-SIINFEKL complexes were generated from GFP-tagged antigen (Fig 4C) were, surprisingly, equivalent in each DC subset (Figs 4B and 4C). This suggests that all DC subsets are equally capable of presenting endogenous antigen when infected.

### CD11b^+^ DC and pDC stimulate antigen specific T_CD8+_ via direct priming, while CD8α^+^ DC utilize both direct and cross-presentation pathways

Because uninfected DC stimulated T_CD8+_ (Fig 2D bottom panel) we asked whether specific uninfected DC subsets were specialized to present antigen. We compared the ability of uninfected and ECTV-infected DC subsets to activate naive OT-I T_CD8+_ following a footpad injection with NP-S-EGFP. Twenty-four h.p.i., D-LN cells were FACS-sorted for EGFP^+^ and EGFP^-^ DC subsets. Isolated DC sub-populations were co-cultured with naïve OT-I T_CD8+_, and 60 h later T_CD8+_ proliferation was determined. Infection with NP-EGFP did not induce proliferation of OT-I T_CD8+_ (Fig 4D). Infected CD11b^+^ DC and pDC from mice infected with ECTV-NP-S-EGFP were highly efficient in stimulating naïve OT-I T_CD8+_, but uninfected CD11b^+^ DC and pDC did not significantly prime T_CD8+_ (Fig 4D). However, both ECTV-infected and uninfected CD8α ^+^ DC activated OT-I T_CD8+_ (Fig 4D), indicating that the T_CD8+_ activation by uninfected DC in Fig 2D was mediated by cross presentation by CD8α ^+^ DC.

The inflammatory milieu and expression of costimulatory molecules can also affect the efficiency of T_CD8+_ stimulation. Therefore, the inability of EGFP^-^ CD11b^+^ DC and pDC isolated from NP-S-EGFP-infected D-LN to prime naïve OT-I T_CD8+_ could be attributed to the lack of or lower expression of co-stimulatory molecules, such as CD80 (B7.1) and CD86 (B7.2), compared to EGFP^+^ DC. However, there was no significant difference in expression of CD86 between ECTV-infected and uninfected DC in any of the subsets examined and only minor changes in CD80 expression in the CD11b^+^ population (Fig 4E). Therefore, ECTV infection of DC does not inhibit maturation and changes in costimulatory molecule expression induced by infection are unlikely to account for the differential ability of uninfected DC subsets to present antigen.

### Maturation of DC by TLR agonist treatment does not enhance direct presentation *in vivo*

**Fig 5:**
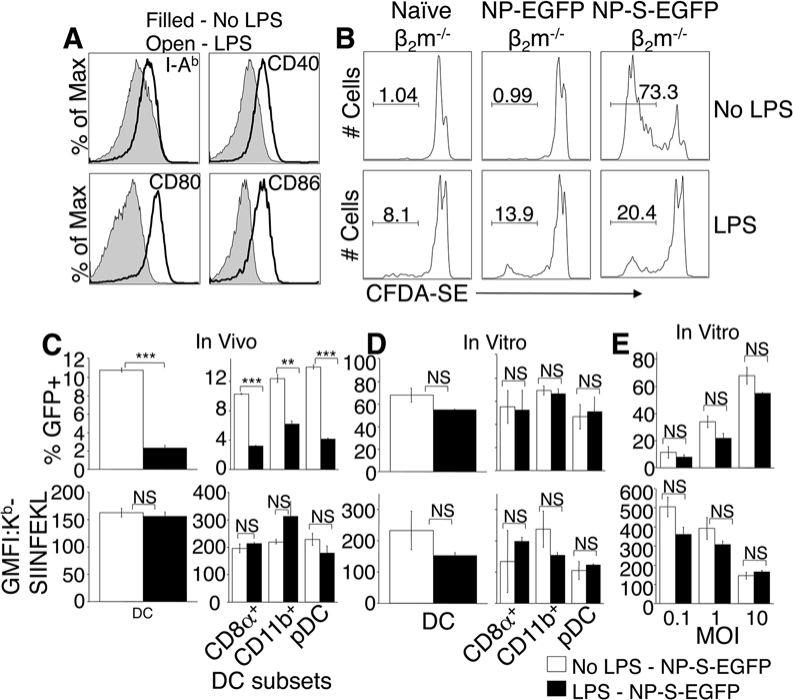
Treatment with TLR agonists *in vivo* inhibits viral infectivity but does not enhance direct antigen presentation. (A) Mice were injected i.v. with LPS, and 12 hr later splenocytes stained to identify DC, and examine expression of MHC class II, CD40, CD80, and CD86. Representative of 3 experiments, using 3 mice per condition. (B) CFDA-SE-labeled OTI T_CD8+_ were adoptively transferred into mice that were then treated with LPS i.v. and 12 hours later injected i.p. with β_2_m^-/-^ cells infected with NP-EGFP or NP-S-EGFP as above. Three days later, OTI T_CD8+_ cell proliferation was determined by CFDA-SE dye dilution. Nos. represent % of cells representative of 3 experiments, using 3 mice per condition. (C) Mice were injected with LPS as above, and 12 h later, the mice were infected i.v with NP-S-EGFP. Twelve h.p.i., splenocytes were stained for DC subsets. Graphs depict ECTV-infection of DC (top panel, left) or DC subsets (top panel, right), and direct presentation by DC (bottom panel, left) or DC subsets (bottom panel, right). Data are pooled from 3 experiments, using 3 mice per condition (mean ± standard error). (D) Splenocytes were harvested and treated with LPS for 12 h, then infected with NP-S-EGFP (MOI = 10). Twelve h.p.i., cells were stained as described in (C). Graphs depict ECTV-infection of DC (top panel, left) or DC subsets (top panel, right), and direct presentation by DC (bottom panel, left) or DC subsets (bottom panel, right). Data are pooled from 3 experiments, using 3 mice per condition (mean ± standard error). (E) Splenocytes were treated with LPS for 12 h, then infected with NP-S-EGFP at the MOI indicated. Graphs depict infection of DC (top panel) and direct presentation by DC (bottom panel). Data are representative of two independent experiments (mean ± standard dev). P values *p<0.05, **p<0.01, ***p<0.001, NS (not significant). Student’s unpaired t-test.

Maturation of DC has been reported to enhance antigen presentation, and systemic *in vivo* activation of DC by TLR agonists such as LPS, CpG-B, and Poly I:C is reported to block cross presentation of viral antigen by uninfected cells [18]. However, TLR ligation inhibited influenza virus infection of DC *in vitro* [19] and markedly reduced *in vivo* viral loads following infection with the poxvirus VACV [20], potentially reducing antigen presentation by infected cells. We asked whether TLR ligation and maturation of pAPC altered infection, antigen production or presentation. As expected, TLR treatment stimulated maturation of DC, following 12 hr CpG-B (not shown) or LPS treatment *in vivo*, as assessed by upregulation of MHC class II (I-A^b^), CD40, CD80 and CD86 (Fig 5A). This 12 hr pre-treatment with TLR ligands also inhibited proliferation of adoptively transferred CFDA-SE labeled OT-I T_CD8+_ following immunization with presentation incompetent β_2_m^-/-^ cells that were infected *in vitro* for 6 hours with either NP-EGFP, NP-S-EGFP or were left uninfected. As β_2_m^-/-^ cells lack MHC class I and therefore cannot present antigen, this indicates that T_CD8+_ priming in this system via cross-presentation is inhibited by systemic TLR ligation (Fig 5B). In contrast, the majority of OT-I T_CD8+_ in untreated mice that received β_2_m^-/-^ cells infected with NP-S-EGFP proliferated (Fig 5B). Therefore, presentation of ECTV-derived antigen by uninfected pAPC was inhibited by TLR agonist treatment *in vivo*.

We next assessed whether TLR agonists affected ECTV infection of pAPC or direct antigen presentation by infected pAPC. Mice were injected with CpG-B, Poly I:C, or LPS, and then infected with either NP-EGFP or NP-S-EGFP. Presentation of antigen by infected pAPC was quantified 12 h.p.i. by flow cytometry. *In vivo* treatment with TLR agonists resulted in an approximate 70% reduction in the numbers of ECTV-infected DC (Fig 5C) and other pAPC (not shown), indicating that DC maturation dramatically reduces virus infection. This inhibition of ECTV infection of DC extended across all the sub-populations examined (Fig 5C, top panels), but GFP fluorescence in the infected population was not altered by TLR ligation (not shown). Examination of antigen presentation by the remaining 30% of infected DC revealed that infected mature DC were able to directly present antigen with the same efficiency as DC that were not exposed to TLR agonists (Fig 5C, lower panels), suggesting that DC maturation did not enhance direct presentation *in vivo*.

To reconcile our findings with those describing a role for DC maturation in enhanced antigen presentation [21, 22], and no effect of TLR ligation upon virus infection *in vitro* [18], we isolated DC from mice, treated with LPS or CpG-B for 12 h, and infected with NP-S-EGFP. TLR ligation prior to virus infection did not inhibit ECTV infectivity of DC or DC subsets *in vitro* (Fig 5D, top panel), regardless of MOI (Fig 5E, top panel). TLR ligation also did not enhance direct antigen presentation (Fig 5D, bottom panel) even when DC were infected at various MOI (Fig 5E, bottom panel). However, at the highest MOI, overall direct presentation was significantly lower, presumably due to ECTV-induced cell death (Fig 5E, bottom panel).

### Cross presentation by uninfected CD8α^+^ DC is not generally required for induction of a T_CD8+_ response

**Fig 6:**
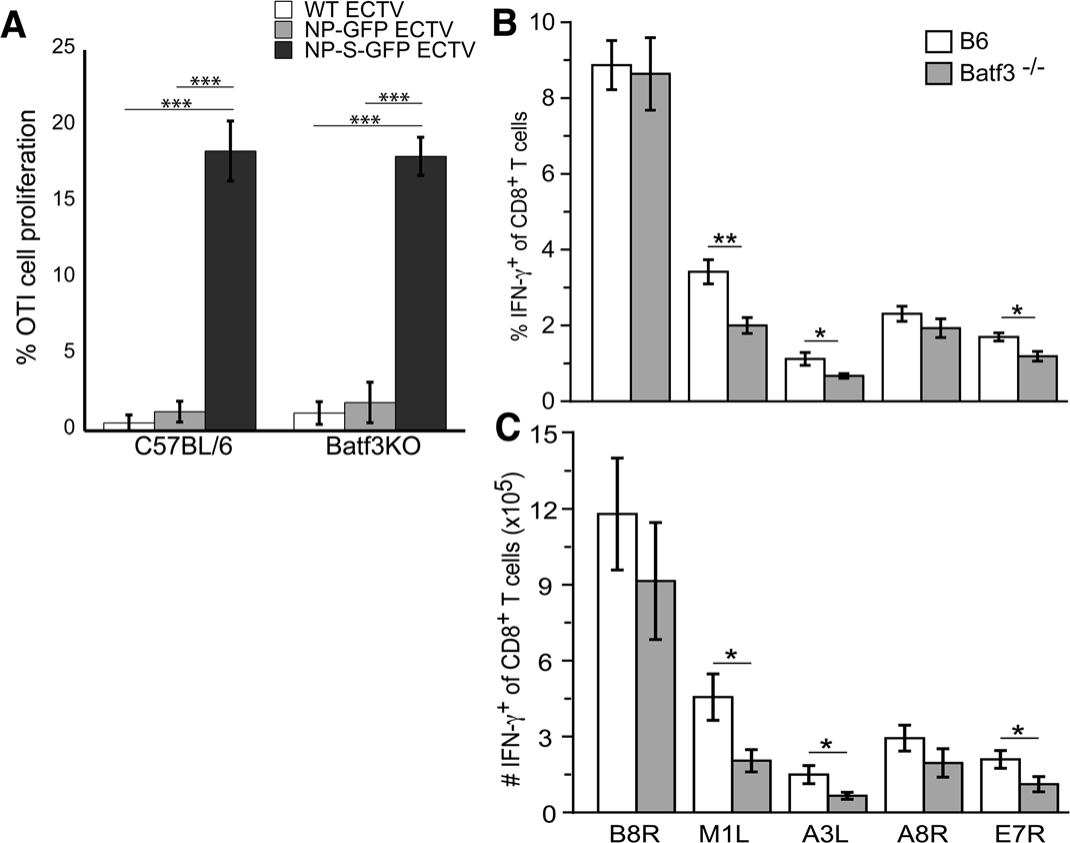
CD8 α^+^ DCs are not required for activation of the T_CD8+_ response to ECTV infection. (A) OT-I T_CD8+_ stained with CFDA-SE were adoptively transferred into B6 or Batf3^-/-^ mice 24 h before infecting i.d. with WT (light gray bars), NP-EGFP (dark gray bars), or NP-S-EGFP (black bars). Two d.p.i., D-LNs were harvested and cells were analyzed for proliferation. Data were pooled from two experiments, using 5 mice per condition (mean ± standard error). The percentage (B) and number (C) of IFN-γ^+^ cells of total T_CD8+_ in response to ECTV-specific peptide stimulation. B6 (white bars) or Batf3^-/-^ (gray bars) mice were infected with WT ECTV i.d. Splenocytes harvested on day 7 p.i. were stimulated *ex vivo* with ECTV-specific peptides and IFN-γ production by T_CD8+_ was assessed by intracellular cytokine staining. Data are representative of 2 experiments using 3 mice per condition (mean ± standard error). P values *p<0.05, **p<0.01, ***p<0.001, NS (not significant). Student’s unpaired t-test.

Our data above indicate that during ECTV infection only CD8α ^+^ DC can present antigen when uninfected. To test the importance of this pathway for induction of antigen-specific T_CD8+_ we infected wild-type or Batf3^-/-^ mice with NP-S-EGFP. Batf3^-/-^ mice lack CD8α ^+^ DC and have a significant defect in cross presentation [23]. At 2 d.p.i. no proliferation of adoptively transferred OT-1 T_CD8+_ was observed in the spleen following infection with either NP-SEGFP or control NP-EGFP (not shown). We did observe proliferation of OT-1 in the D-LN after infection with NP-S-EGFP (Fig 6A), but the proliferation observed was equivalent in wild-type and Batf3^-/-^ mice, indicating that CD8α ^+^ DC are dispensable for initiation of an OVA-specific T_CD8+_ response.

To extend our observation beyond an OVA-specific response and beyond the use of the highly sensitive OT-1 TCR T_CD8+_ we examined the functional activation of T_CD8+_ specific for native ECTV encoded epitopes within the B8R, M1L, A3L, A8R, and E7R viral proteins. Seven days after infection, the frequency (Fig 6B) and numbers (Fig 6C) of T_CD8+_ producing IFN-γ in response to the B8R and A8R epitopes were equivalent in wild-type and Batf3^-/-^ mice. However, responses to the M1L, A3L, and E7R epitopes were reduced in Batf3^-/-^ mice (Figs 6B and6C), indicating that presentation by CD8α ^+^ DC may be required for maximal presentation of some determinants.

## Discussion

Vaccines aimed at inducing protective T_CD8+_ responses have the promise of targeting invariant intracellular proteins that can be used to clear the pathogens encoding the antigens when antibody responses are ineffective. Recent studies have indicated that pAPC, and particularly DC, subpopulations are specialized to induce T cell responses via different antigen presentation pathways. Recent vaccine strategies have specifically targeted exogenous antigen to particular DC populations, often along with ligands known to induce DC maturation, in an attempt to increase the efficacy of T_CD8+_ priming [7]. However, our work reveals that for vaccines aimed at inducing protective T_CD8+_, targeting only individual pAPC populations, particularly with exogenous antigens, may drastically reduce the presentation of peptide-MHC complexes *in vivo*, irrespective of DC maturation. In particular, our results indicate that the number of peptide MHC complexes generated from endogenous sources dramatically outnumbers those produced from exogenous sources. Indeed, peptide-MHC complexes produced from exogenous sources were below the level of detection using our specific antibody (>100 complexes per cell [14]) even when mice were immunized with 3 x 10^7^ infected cells expressing large quantities of viral protein. Therefore it is clear that if a virus infects a pAPC more peptide-MHC complexes are likely to be produced than if these cells remain uninfected, even if targeted exogenously. This finding may have been hidden by the experimental use of mismatched human virus/murine target combinations where virus tropism is diverted away from pAPC, which are often a Trojan Horse when infected that allow transmission of numerous viruses. The use of the ECTV system reveals that during a fulminant natural infection, direct presentation likely predominates during induction of protective T_CD8+._

Increasing the number of pMHC-I on the surface of an APC *in vitro* causes activation induced cell death and allows survival of only low affinity T_CD8+_ [24]. In contrast, increasing the number of pMHC-I *in vivo* can increase the number of T_CD8+_ primed [25, 26] up to a certain point [27], and does not reduce the affinity of the responding T_CD8+_. Therefore, a vaccine vector that produces a larger number of cell surface pMHC-I will produce more effective T_CD8+_. The T_CD8+_ response to the poxvirus VACV is initiated following antigen presentation by infected APC [16, 20, 28]. Here we demonstrate that the number of pMHC-I presented by infected pAPC vastly outnumbers the number of complexes presented by uninfected pAPC, even when the antigen is readily available for presentation by both infected and uninfected cells. Therefore, our findings show that the most efficient way to induce a strong T_CD8+_ response is to utilize a vaccine in which endogenous expression of antigen within pAPC is optimized.

Here we found that uninfected CD8α^+^ DC were able to present exogenously derived viral antigen. Previous studies have implicated CD8 α^+^ DC in the presentation of all viral antigen [1], but these studies may reflect preferential infection of certain DC subpopulations by viruses [29], or exclusive presentation of exogenous antigen as pAPC are not infected [2, 3]. In addition, it has been proposed that some pAPC populations are specialized to present peptides on MHC Class I while other populations are specialized to present on MHC Class II [5]. Support for this hypothesis comes from gene array analysis describing a paucity of expression of components of the MHC Class I processing pathway in DC populations that did not present exogenous antigen [5]. Importantly, these studies only examined presentation of exogenous antigen. Virtually all nucleated cells express both MHC Class I and the machinery required to present peptide-MHC complexes derived from endogenous antigens. Specialization of pAPC populations to avoid such presentation would furnish viruses and intracellular bacteria with a location in which they could replicate with relative indifference to the action of the adaptive immune system. Therefore, it is logical that all infected pAPC will present pMHC-I derived from endogenous antigens, and this is indeed what we observe.

We examined the relative efficiency of presentation of endogenous antigens to reveal that DC do not appear to be more efficient at presenting endogenous antigens than B cells, although both appear to be better than macrophages (Fig 2C). There is no specialization within DC subpopulations, a pronounced difference from the presentation of exogenous antigens, which CD8α^+^ DCs are substantially superior at presenting [2]. This lack of specialization by DC populations is at odds with the gene array data indicating differential expression of MHC Class I processing machinery [5, 30]. However, the supply of antigenic peptide, rather than the expression of any processing components, is limiting in MHC Class I presentation [31]. Therefore, the rate of antigen production and degradation controls the efficiency and amplitude of antigen presentation in infected cells. In the system examined here, DC (of all subsets) produce more fluorescent antigen than other pAPC, and so present a higher number of peptide-MHC complexes per cell. Peptides are generated from endogenous short-lived proteins, termed Defective Ribosomal Products (DRiP) or Rapidly Degraded Proteins (RDPs) [22, 32] much more efficiently than from long-lived proteins, which are the substrates for cross presentation [33]. DRiP/RDP are unlikely to be correctly folded and therefore may not be fluorescent in our system. Our calculations of the relative efficiency of antigen presentation are made with the assumption that the proportion of newly synthesized protein within the RDP fraction is equal between pAPC populations. There are no publications that indicate the contrary.

DC that were ECTV-infected following TLR agonist treatment directly presented antigen at equivalent levels to untreated DC, demonstrating that DC maturation does not enhance antigen presentation and so likely does not affect the supply of antigenic peptide. Systemic TLR ligation did block cross presentation, as previously published [18], but it also reduced ECTV infection by around 70% demonstrating that, as with VACV infection, TLR ligation fails to differentiate between antigen presentation by infected and uninfected pAPC [20]. Pre-treatment of DC with TLR ligands rendered DC resistant to influenza virus infection *in vitro* [19]. However, we did not observe a decrease in virus infectivity when DC were treated with TLR agonists *in vitro*, regardless of MOI. It is possible that this may reflect an overall reduction in DC infectability that is a byproduct of the DC isolation procedure upon infectability with ECTV, but this is unavoidable. Nonetheless, we did not observe an inhibition of infection by TLR treatment in vitro. Thus, systemic TLR ligation may reduce the infectability of pAPC populations via an indirect mechanism, such as the relocalization of DC populations, alteration in virus drainage to reduce cellular exposure to virus, or inhibition of virus replication through induction of innate antiviral pathways.

Using current methodology, it has not been possible to differentiate between infection of DC in the periphery or in the D-LN. However, at early time points following ECTV infection i.d, the ECTV-infected cells in the D-LN were found predominantly below the sub-capsular sinus, and phenotypic analysis showed that these infected cells were CD169^+^ macrophages. Infection of macrophages found within or below the sub-capsular sinus has been previously reported with VACV and vesicular stomatitis virus infection [16, 17, 34]. Our kinetic studies of ECTV infection revealed that macrophages were probably the first pAPC to be infected by 6 h.p.i., while B cells and DC were infected by 12 hours post-ECTV infection (S2 Fig). These findings suggest that virus drained from the site of infection into the D-LN and subsequently infected DC, although we cannot exclude the possibility that ECTV-infected DC migrated from the site of infection into the D-LN at later time points [35].

Although it was expected that only certain infected pAPC populations interact with naïve T_CD8+_ we readily identified naïve T_CD8+_ interacting with all of the pAPC populations that are presenting antigen. The interaction of macrophages and DC with T_CD8+_ during a poxvirus infection has been previously described [16, 17]. Previous reports also showed that recently triggered antigen-specific T_CD8+_ relocated to the peripheral regions in an area termed the “peripheral inter-follicular region” [17]. This region was just below the LN sub-capsular sinus, and T_CD8+_ were shown to interact with DC found in this macrophage-rich region of the LN. Interaction with infected macrophages may induce an intermediate activation phenotype [17]. The rapid decline in GFP^+^ cells following VACV infection indicates that this non-native virus infection rapidly kills the cells that it infects and inefficiently infects other cells in the D-LN, which contributes to our inability to purify significant numbers of VACV-infected cells [16, 36]. All ECTV-infected pAPC populations (including infected B cells) purified from infected mice were able to trigger *in vitro* proliferation of naïve T_CD8+_, and interact with naïve T_CD8+_ *in vivo*. The interaction of infected B cells and naïve T_CD8+_ observed is surprising, the separation between the T cell zone and the B cell follicle within secondary lymphoid organs is carefully regulated by tightly controlled chemokine gradients. However, poxviruses, including ECTV, encode chemokine-binding proteins [37] that likely alter the balance of local chemokines in infected LN. Such an alteration in local chemokine gradient could allow interaction of T_CD8+_ with infected B cells. Notably, very few T_CD8+_ were visualized in the B cell follicles but were mainly distributed in the cortical region and marginal zones of the LN. This suggests that ECTV-infected B cells may have migrated to the inter-follicular regions where they interacted with antigen-specific T_CD8+_. Our ongoing efforts seek to understand the impact of ECTV-mediated changes in local chemokine gradients on the role of B cells in induction of ECTV-specific T_CD8+_ and T_CD4+_.

Overall, our results are of importance for both vaccine design and to appreciate the basic mechanisms responsible for induction of a T_CD8+_ response to a fulminant widespread virus infection. In a vaccine the most effective way to induce large numbers of antigen-specific T_CD8+_ appears to be expression of antigen endogenously within pAPC populations, as the number of peptide-MHC complexes generated from endogenous antigens far exceeds those produced from exogenous sources. Specific DC populations did not display enhanced presentation capabilities, and prior induction of a T_CD8+_ response did not enhance antigen presentation on a cellular level. Our data indicate that a viral vector that effectively infects multiple pAPC populations and induces an inflammatory state via expression of natural pattern recognition receptor ligands may induce an optimal protective T_CD8+_ response. In terms of the basic mechanisms responsible for induction of a T_CD8+_ response it appears that a widespread natural infection may primarily use direct presentation by infected pAPC to prime naïve T_CD8+_. The predominance of the use of cross presentation in the literature may be a byproduct of the study of human viruses in the mouse or of viruses that specifically avoid infection, even if unproductive, of pAPC populations.

## Materials and Methods

### Mice

C57BL/6 mice were purchased from Charles River Laboratories. Beta 2-microglobulin (β_2_m^-/-^) [38], OT-I [15], TAP1^-/-^ [39] were from Jackson and were bred and housed in the specific-pathogen-free animal facility at the Hershey Medical Center. The Penn State College of Medicine Institutional Animal Care and Use Committee approved all studies.

### Viruses

Recombinant ECTV (Moscow strain) encoded a fusion protein consisting of the influenza virus A/NT60 nucleoprotein (NP) affixed to the NH_2_-terminus of enhanced green fluorescent protein (EGFP) [12]. Ovalbumin (OVA) residues 257–264 (SIINFEKL) were inserted between the NP and EGFP to produce NP-S-EGFP. A control virus that lacks SIINFEKL peptide is denoted as NP-EGFP. Replication of each recombinant virus *in vitro* and *in vivo* is similar to wild-type ECTV. Mice were immunized with 10^6^ plaque-forming units (PFU) of rECTV intravenously (i.v.), intraperitoneally (i.p.), intradermally (i.d) in the ear pinnae, or footpad injection. For *in vitro* studies, cells were infected with ECTV at a multiplicity of infection (MOI) of 0.1, 1 or 10, depending on the experiment.

### Toll-like receptor (TLR) agonist treatment

*In vivo*, mice were injected i.v., and *in vitro*, splenocytes were treated with 15 μg/ml of Escherichia coli 055:B5 lipopolysaccharide (LPS) (Sigma-Aldrich), 20 μg/ml of CpG-B oligonucleotides 1826 (Invivogen) and 20 μg/ml of Polyinosinic:polycytidylic acid (Poly I:C) (Sigma-Aldrich) dissolved in phosphate buffered saline (PBS).

### Isolation of T_CD8+_ from OT-I.SJL transgenic mice

Spleens and lymph nodes were harvested from OT-I.SJL mice and cells incubated with anti-CD8α beads, and T_CD8+_ were positively selected using an AutoMACS sorter (Miltenyi Biotech). To assess T_CD8+_ proliferation, Carboxyfluorescein diacetate, succinimidyl ester (CFDA-SE) (Invitrogen) labeled OTI.SJL T_CD8α+_ cells were adoptively transferred into mice on day minus 3 by i.v. injection into the tail vein. On day 3, T_CD8+_ cell proliferation was determined by dilution of CFDA-SE fluorescence using flow cytometry. For visualization, T_CD8+_ were labeled with 5 μM CellTracker Orange CMTMR (5-(and-6)-(4-chloromethyl)benzoyl)amino)tetramethylrhodamine (Invitrogen) and adoptively transferred into mice. Twenty four hours later, the mice were infected with rECTV, and the draining lymph nodes (D-LN) were harvested and frozen. Cryostat sections (30 μm) were cut and fixed in 4% paraformaldehyde.

### Immunofluorescence microscopy

Cryostat sections were incubated with F_ab_ donkey anti-mouse IgG (Jackson ImmunoResearch) then stained with directly labeled APC-conjugated anti-CD11c (N418) (eBiosciences) or Alexa-647 conjugated anti-B220/CD45R (RA3-6B2) (eBiosciences) antibodies. Staining with the unlabeled primary antibodies anti-CD103 (BioLegend) or anti-CD169 (3D6.112) (Serotec) was revealed by staining with Cy-5 conjugated F_(ab)2_ donkey anti-rat IgG (Jackson ImmunoResearch). Staining was visualized using an Olympus 1X81 deconvolution microscope and Slidebook 5.0 digital microscope.

### Flow cytometry

Antibodies to the following molecules were purchased from eBioscience unless otherwise stated: MHC class II (I-A^b^) (25-9-17), CD11c (N418), CD45.1 (A20), CD80 (16-10A1), CD45R/B220 (RA3-6B2), CD19 (ID3), NK1.1 (PK136), CD90.2 (53-2.1), CD11b (M1/70), CD8α (53-6.7), Streptavidin, CD86 (GL1) (BD Biosciences), CD40 (3/23) (BD Biosciences), CD169 (3D6.112) (Serotec), and 25-D1.16 (grown, purified and labeled in house).

### UV/psoralen treatment of viruses and gamma-irradiation of cells

β_2_m^-/-^, STBKM-1 fibroblast cells or C57BL/6.SJL lymphoid cells were infected with ECTV at an MOI = 10 for 6 hours, then treated with psoralen and UV-C light (254 nm) for 1 hour, as previously described [13]. The mice were then administered LPS i.v. on day 0, then 12 hours later injected i.p. with UV-treated/gamma-irradiated β_2_m^-/-^ cells that were infected with NP-EGFP or NP-S-EGFP.

### T_CD8+_ cell stimulation by *ex vivo* isolated pAPC

Using a MoFlo XDP cell sorter, popliteal lymph node cells were sorted for EGFP^+^ or EGFP^-^ pAPC: Macrophages (CD11c^-^CD19^-^B220^-^CD11b^+^CD169^+^), B cells (CD11c^-^CD11b^-^CD169^-^CD19^+^B220^+^), DC (CD19^-^NK1.1^-^CD90^-^CD11c^+^), and DC subsets (CD8α^+^B220^-^CD11b^-^, CD11b^+^CD8α^-^B220^-^, B220^+^CD11b-CD8α^-^). Cells were co-cultured with OTI.SJL T_CD8+_ at 1:8 DC:T cell ratio for 60 hours, then proliferation of OTI.SJL T_CD8+_ measured by flow cytometry. To prevent T cell infection by ECTV 50 μM Vistide/Cidofovir (Gilead) was added.

### *Ex vivo* intracellular cytokine staining (ICS) assay

Spleens were harvested from B6 and Batf3^-/-^ mice at 7 days post infection (d.p.i.) with ECTV, and cells stimulated for 5 hrs with 1 μg/mL of ECTV-specific peptide (B8R_20-27_ (TSYKFESV), M1L_424-438_ (KSIIIPFIAYFVLMH), A3L_270-277_ (KSYNYMLL), A8R_189-196_ (ITYRFYLI) and E7R_130-137_ (STLNFNNL)) or no peptide in the presence of 10 μg/mL of brefeldin A. After stimulation, cells were washed, fixed in 2% paraformaldehyde and permeabilized prior to staining intracellularly for IFN-γ. Net frequencies and numbers of epitope-specific T_CD8+_ were calculated by subtracting the no peptide background response.

## Supporting Information

S1 Fig (A) ECTV infection is dependent on virus replication. Mice were injected i.d. with vehicle, NP-EGFP or UVC/psoralen inactivated NP-EGFP. Twenty-four hours post infection, cervical LN were harvested and EGFP^+^ cells were analyzed by flow cytometry. (B) Gating strategy to identify ECTV-infected pAPC. Mice were injected i.d. with vehicle, NP-EGFP or NP-S-EGFP i.d., and D-LN were harvested at 24 h.p.i. Cells were stained with antibodies to identify pAPC as: DC (CD11c^+^ CD169^−^ CD19^−^), macrophages (CD169^+^ CD11b^+^ CD11c^−^ CD19^−^), and B cells (CD19^+^ B220^+^ CD11c^−^ CD169^−^). Numbers represent percentage of cells.
(DOCX)

S2 Fig (A) Kinetic analysis to determine when pAPC become infected by ECTV. Mice were injected with NP-EGFP i.d. and cervical LN harvested at various time points post infection. EGFP^+^ pAPC were assessed following staining with antibodies to identify pAPC as outlined in S1B Fig. (B) K^b^-SIINFEKL complexes on the surface of each population of pAPC. Mice were injected with NP-S-EGFP i.d. and D-LN harvested at various time points post infection. pAPC were identified as described above, and GMFI of 25-D1.16 was performed to quantify levels of K^b^-SIINFEKL complexes on EGFP^+^ pAPC.
(DOCX)
