# Supplementary material for: Peptide-MHC-I from Endogenous Antigen Outnumber Those from Exogenous Antigen, Irrespective of APC Phenotype or Activation
Source: PLoS Pathog. 2015 Jun 24;11(6):e1004941. doi: 10.1371/journal.ppat.1004941 (PMC4479883; doi:10.1371/journal.ppat.1004941)
Supplement: S1 Fig — Mice were injected i.d. with vehicle, NP-EGFP or UVC/psoralen inactivated NP-EGFP. Twenty-four hours post infection, cervical LN were harvested and EGFP+ cells were analyzed by flow cytometry. (B) Gating strategy to identify ECTV-infected pAPC. Mice were injected i.d. with vehicle, NP-EGFP or NP-S-EGFP i.d., and D-LN were harvested at 24 h.p.i. Cells were stained with antibodies to identify pAPC as: DC (CD11c+ CD169− CD19−), macrophages (CD169+ CD11b+ CD11c− CD19−), and B cells (CD19+ B220+ CD11c− CD169−). Numbers represent percentage of cells. (DOCX) [file ppat.1004941.s001.docx]

**
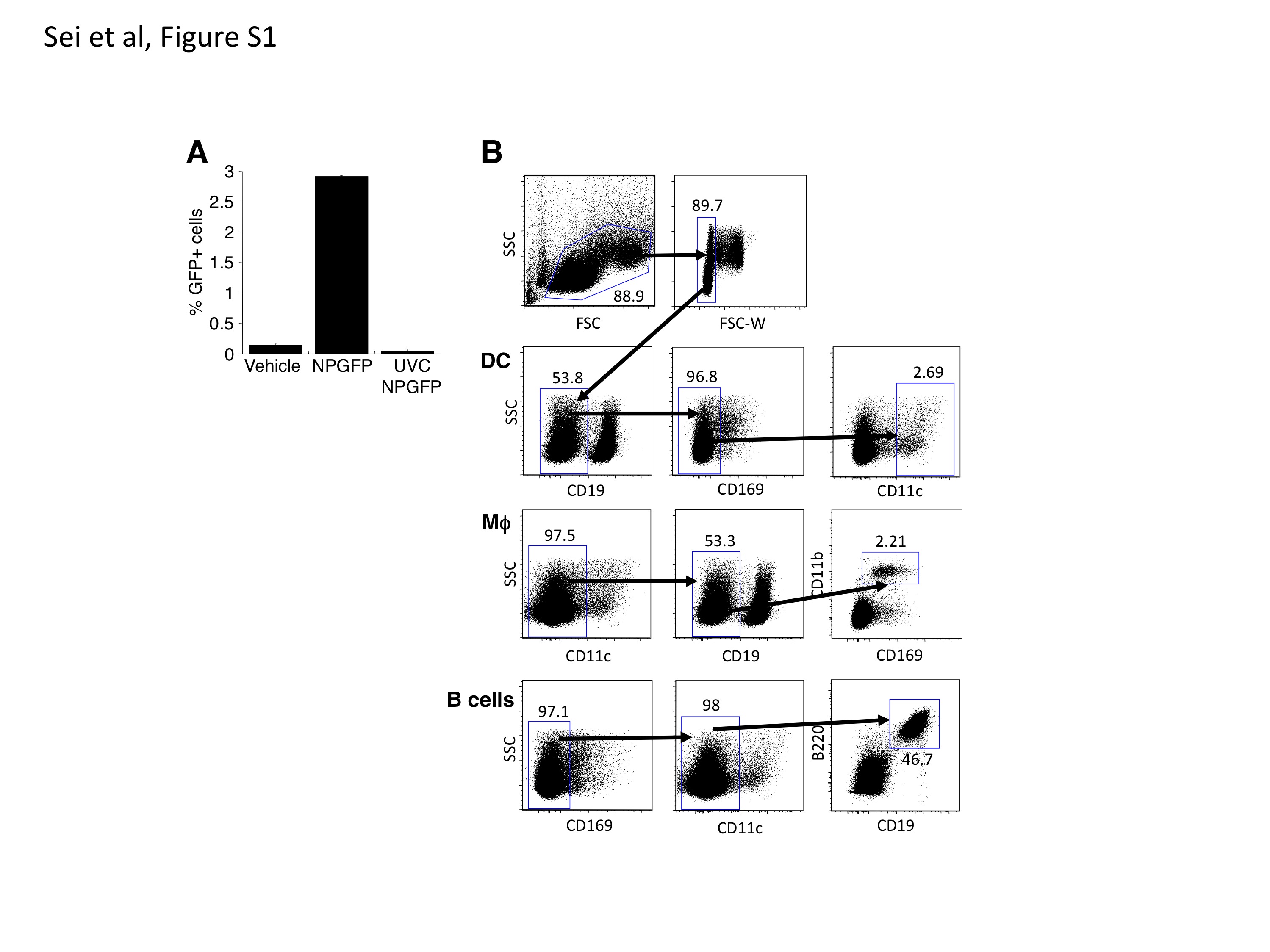
**

**Supplementary Figure 1.**

(A) ECTV infection is dependent on virus replication. Mice were injected i.d. with vehicle, NP-EGFP or UVC/psoralen inactivated NP-EGFP. Twenty-four hours post infection, cervical LN were harvested and EGFP^+^ cells were analyzed by flow cytometry. (B) Gating strategy to identify ECTV-infected pAPC. Mice were injected i.d. with vehicle, NP-EGFP or NP-S-EGFP i.d., and D-LN were harvested at 24 h.p.i. Cells were stained with antibodies to identify pAPC as: DC (CD11c^+^ CD169^−^ CD19^−^), macrophages (CD169^+^ CD11b^+^ CD11c^−^ CD19^−^), and B cells (CD19^+^ B220^+^ CD11c^−^ CD169^−^). Numbers represent percentage of cells.
