# Supplementary material for: Peptide-MHC-I from Endogenous Antigen Outnumber Those from Exogenous Antigen, Irrespective of APC Phenotype or Activation
Source: PLoS Pathog. 2015 Jun 24;11(6):e1004941. doi: 10.1371/journal.ppat.1004941 (PMC4479883; doi:10.1371/journal.ppat.1004941)
Supplement: S2 Fig — Mice were injected with NP-EGFP i.d. and cervical LN harvested at various time points post infection. EGFP+ pAPC were assessed following staining with antibodies to identify pAPC as outlined in S1B Fig. (B) Kb-SIINFEKL complexes on the surface of each population of pAPC. Mice were injected with NP-S-EGFP i.d. and D-LN harvested at various time points post infection. pAPC were identified as described above, and GMFI of 25-D1.16 was performed to quantify levels of Kb-SIINFEKL complexes on EGFP+ pAPC. (DOCX) [file ppat.1004941.s002.docx]

**
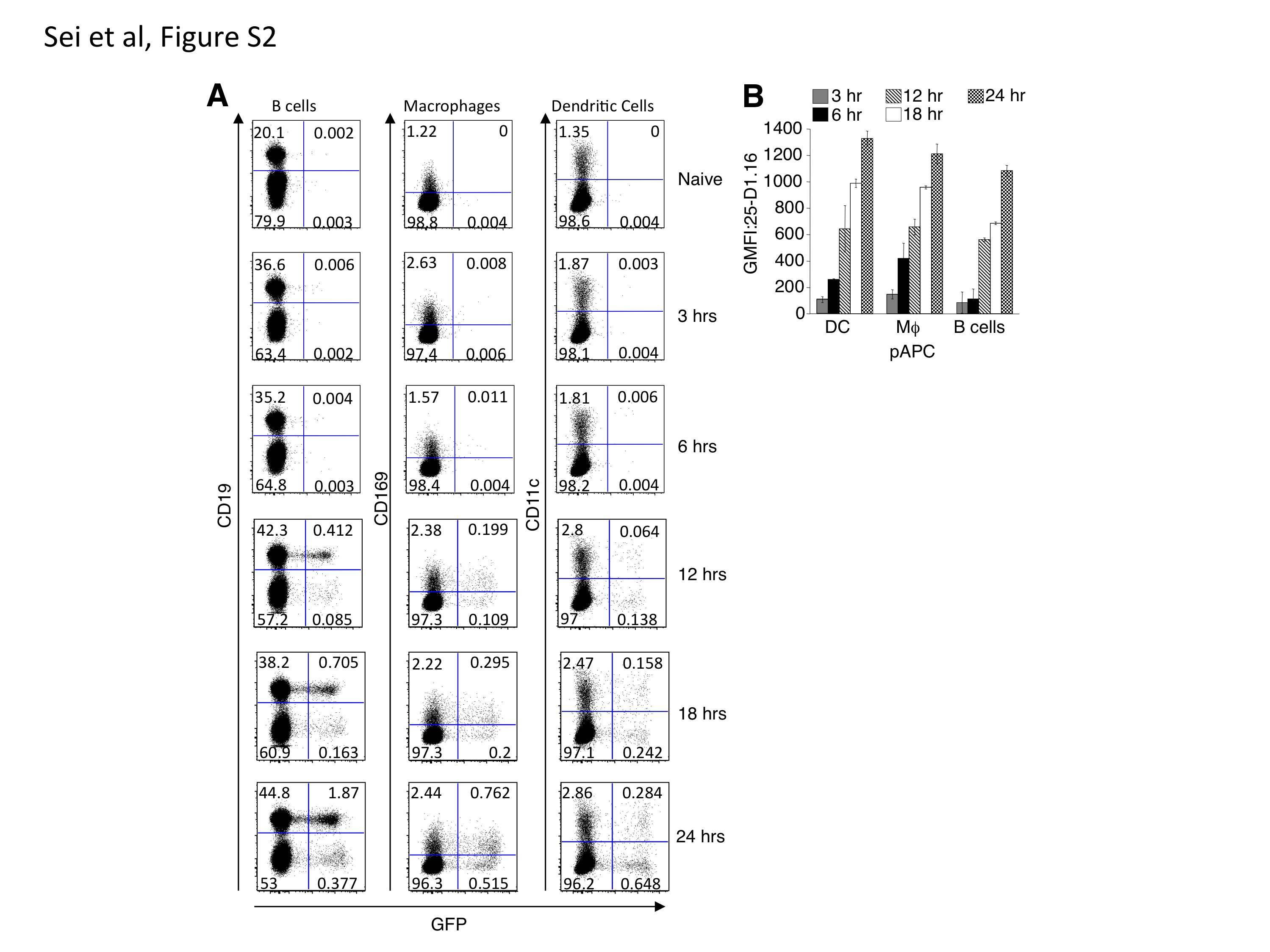
**

**Supplementary Figure 2.**

(A) Kinetic analysis to determine when pAPC become infected by ECTV. Mice were injected with NP-EGFP i.d. and cervical LN harvested at various time points post infection. EGFP^+^ pAPC were assessed following staining with antibodies to identify pAPC as outlined in Fig. S1B. (B) K^b^-SIINFEKL complexes on the surface of each population of pAPC. Mice were injected with NP-S-EGFP i.d. and D-LN harvested at various time points post infection. pAPC were identified as described above, and GMFI of 25-D1.16 was performed to quantify levels of K^b^-SIINFEKL complexes on EGFP^+^ pAPC.
